# Dermoglandular advancement-rotation flap for conservative treatment of breast cancer – description of technique, objective and subjective assessments

**DOI:** 10.3389/fonc.2023.1137924

**Published:** 2023-05-03

**Authors:** Maria Carolina Soliani Bastos, Fábio Bagnoli, José Francisco Rinaldi, Thais Businaro Fernandes João, Vilmar Marques de Oliveira

**Affiliations:** Gynecology and Obstetrics Department, School of Medical Sciences, Santa Casa de Misericórdia de São Paulo, Mastology Section, São Paulo, SP, Brazil

**Keywords:** breast-conservative surgery, breast neoplasms/surgery, breast reconstruction/methods, aesthetics, software, assessment of results/methods

## Abstract

**Objective:**

to describe and evaluate the dermoglandular advancement-rotation flap with no contralateral surgery as a technique for the conservative treatment of breast cancer when skin or a large proportion of gland requires resection.

**Patients/Methods:**

14 patients with breast tumors with a mean size of 4.2 cm and need for skin resection. The resection area is included within an isosceles triangle, with its apex located on the areola, which is the pivot for rotation of a dermoglandular flap released through a lateral extension along that triangle base. Symmetry before and after radiotherapy was objectively assessed by authors using the BCCT.core software, as well as subjectively by three experts and patients themselves using the Harvard scale.

**Results:**

Experts considered the breast symmetry results to be excellent/good for 85.7% of patients in the early post-operative period and 78.6% in the late post-operative period. Excellent/good ratings provided by BCCT.core software amounted to 78.6% of cases in the early post-operative period and 92.9% in the late post-operative period. Symmetry was rated as excellent/good by 100% of patients.

**Conclusion:**

Dermoglandular advancement-rotation flap technique with no contralateral surgery provides good symmetry when a large proportion of skin or gland requires resection on breast conservative cancer treatment.

## Introduction

Breast-conservative surgery is the standard surgical treatment for most breast cancer cases. As demonstrated by solid and long-term follow-up studies, breast-conservative surgery associated with radiotherapy provides overall survival rates comparable to those of radical treatment, and even more recent studies suggest that breast-conservative surgeries may provide higher disease-free survival rates than radical mastectomies (1–9).

Various studies have reported breast asymmetries deriving from conservative surgery, especially when resections exceed 20% of breast volume. When located in the medial, inferior or central quadrants, resection volumes of more than 10% can already produce asymmetries (10–12). Numerous factors contribute to the risk of breast asymmetry, including younger age, high BMI, large tumors, unfavorable tumor location, compromise of skin, need for new surgeries, postoperative seroma and adjuvant radiotherapy (13, 14). An estimated 30% of all women undergoing locoregional treatment experience fair/poor esthetic results, which negatively impacts their psychosocial recovery and quality of life (10, 15).

It is necessary to resect the skin when it is compromised by the tumor or when the skin flap resulting from resection with appropriate margins would result very thin, and therefore, prone to necrosis. When necessary, resection of the skin overlying small tumors in breast-conserving surgeries can be performed using classical techniques, i.e. closing the resected area by approximation of the skin and glandular tissue. In medium to large tumor resections, however, this closure can lead to significant distortions of breast architecture and position of the nipple-areola complex and may require a contralateral mammoplasty as an attempt to achieve some breast symmetry. Resection and remodeling techniques focused on minimizing these distortions may allow for simpler and unilateral surgeries, which would help to save patients’ biological resources – an advantage that is especially beneficial for patients in poor clinical condition. Furthermore, faster and more resolutive surgeries would preserve resources from health services, which may be already overwhelmed and incapable of providing proper care to all patients with breast cancer.

Burow’s Triangle technique was first described in 1855 by Karl August von Burow as a procedure for facial reconstructions (16). This technique consists in releasing a full-thickness flap from adjacent tissues for large-size advancements. This principle can also be used for breast remodeling surgeries after quadrantectomies and involves the advancement-rotation of a full-thickness flap with its pivot centered on the nipple-areola complex. This approach uses adjacent breast tissue to close the resected area, minimizing the nipple’s position distortion. However, an evaluation of the application of this method for breast cancer treatment seems to be unavailable in the literature.

### Objective

The purpose of this study is to describe the surgical technique of dermoglandular advancement-rotation flap for breast remodeling as a breast-conserving treatment of breast cancers, avoiding the need for contralateral surgery, and to evaluate the breast symmetry results by means of objective and subjective assessments.

## Patients and method

This study enrolled 14 patients diagnosed with invasive breast carcinoma who were treated at the Hospital da Santa Casa Misericórdia (São Paulo) between 2016 and 2020. The inclusion criteria for this study were: women older than 18, having breast cancer diagnoses that required the resection of skin that was compromised by the tumor or near it, and which underwent breast remodeling surgery using the dermoglandular advancement-rotation flap technique.

This study has been approved by the Research Ethics Committee of the Santa Casa de São Paulo and participating patients signed an informed consent.

### Surgery

None of the selected patients had any contraindication to breast-conserving surgery. The axillary approach was performed using the same incision that was made for the breast surgery. Patients with clinically negative axillae underwent a biopsy of sentinel lymph node identified after the periareolar injection of 2 ml of blue dye. Axillary dissection was only performed in cases where the axilla was clinically positive or the patient had undergone neoadjuvant chemotherapy and had residual lesions under evaluation using the sentinel lymph node frozen section method.

Vacuum drains were used in all patients until flows amounted to less than 30 ml in 24 hours. No patients had any surgery-related complications, such as hematomas, surgical wound infections and dehiscence. No patients required reoperation.

### Surgical site marking

**Figure 1:**
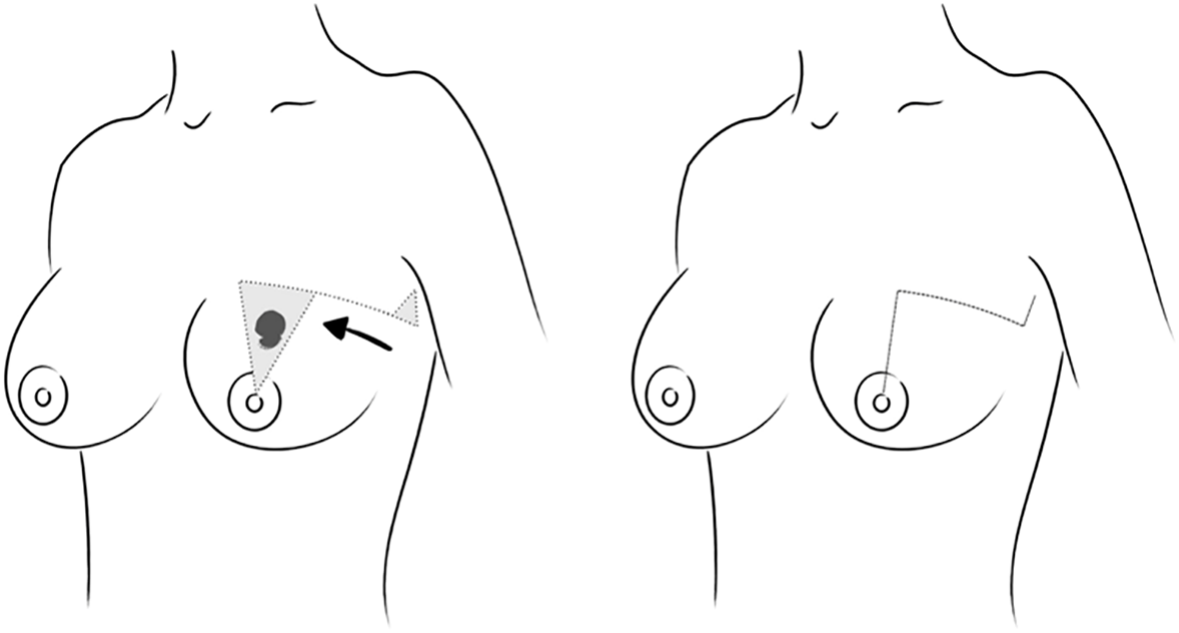
Pre-surgical marking and tissue movement to be performed using a dermoglandular advancement-rotation surgical technique and final appearance.

In surgical site marking, the resection area is delimited with an isosceles triangle with its apex located on the papilla, which will serve as the pivot for rotation of the flap. The base of this triangle is extended laterally. Another isosceles triangle is subsequently marked at the end of this lateral extension. Smaller in size, the apex of this triangle is located on the opposite side of the first triangle. This second triangle demarcates the resection that will be used to correct the excess tissue that the flap advancement will produce. The minimal distance between both triangles must be equal to the base of the first triangle. Marking this second triangle is not mandatory, since sometimes it will also be possible to compensate the excess tissue after advancing the full-thickness flap with no further resection (**Figure 1**).

### Description of surgical technique

**Figure 2:**
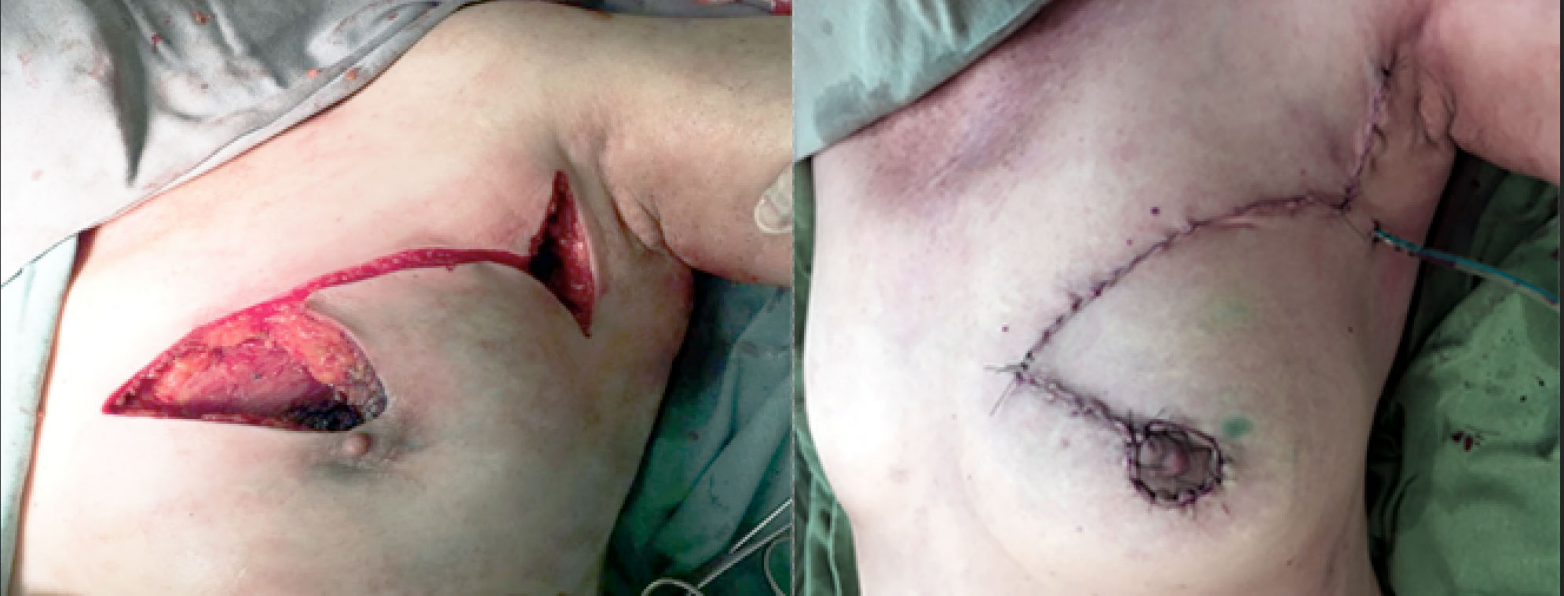
Dermoglandular flap released and before tissue advancement and final surgical appearance after advancement- rotation of dermoglandular flap to correct a defect generated by segmental resection with adjustment of the shape of the nipple-areola complex.

After general anesthesia, the patient lies in the supine position with the arm adjacent to the side that will be operated open at 90 degrees, supported by an arm board. The full-thickness triangle, limited posteriorly by the pectoralis muscle and containing the tumor, is resected using a cold scalpel for the skin and an electric cautery for the gland. Resection is performed so as to provide free margins both macroscopically as well as for the frozen section. Following the previously described quadrantectomy, the extension of the triangle base is incised in full thickness, delimiting a dermoglandular flap that is released from the pectoralis muscle and subsequently advanced and reattached in order to close the resection. This repositioning causes a redundancy of the tissue that was not advanced. This can be corrected by resection of the second triangle or alternatively by distributing the excess tissue along the closure of the incision. Occasionally, it may be also necessary to further adjust the areola’s position, a procedure that is carried out by demarcating the areola with an areola marker and de-epithelizing the adjacent skin to produce a round and properly positioned areola (**Figure 2**)

### Assessment of results

All patients were assessed using photographs taken in the pre-operative, initial post-operative (6-15 days after surgery) and late post-operative (at least 30 days after the end of radiotherapy) stages. The aesthetic results particularly with regards to symmetry were assessed by two mastologists and a plastic surgeon, and rated by them using the Harvard scale in Excelent: when the operated breast is very similiar to the contralateral breast, Good: when the operated breast presents small differences compared to the contralateral breast, Fair: when the operated breast presentes a clear difference, but without serious distortion or Poor: when the operated breast presentes serious distortions when compared with contralateral breast (17). Patients were also requested to rate their results using the same scale. The objective tool chosen for symmetry evaluation was the Breast Cancer Conservative Treatment software Cosmetic results - BCCT.core (18–23).

### Statistical analysis

Statistical analyses included central tendency and dispersion values of the study’s quantitative variables, as well as absolute and relative frequencies of its categorical variables.

Ratings provided by the examiners and the software were grouped into the excellent/good or fair/poor categories for further agreement analyses to be carried out between the different operators and different scales.

This study analyzed the agreement between assessments provided by a panel of experts using the Harvard scale (in the early and late post-operative periods). Agreement between the ratings from experts and the BCCT.core software in both periods was also assessed. The tool used for this assessment was the Kappa coefficient of agreement and its respective 95% confidence intervals and included the subsequent categorization of the coefficients as per the criteria established by Landis & Koch.

The variation between subsequent assessments, i.e., the comparison between the early and late post-operative periods, was analyzed using the McNemar test, which enables the assessment of “before” and “after” situations in which each patient serves as his/her own matched control.

All statistical tests used an alpha error of 5%, in other words, the results were considered to be statistically significant when p<0.05.

## Results

Analyzing the 14 patients, we found that their ages ranged from 42 to 67 years (mean = 58.9 years, standard deviation = 8.4 years). Tumor size ranged from 2 to 7 cm (mean=4.2 cm, standard deviation =1.6 cm). The mean follow-up time for patients was 21.7 months (standard deviation 9.8 months). All patients underwent radiotherapy with a fractionated 46Gy dose + 14Gy boost in 5 - 5.5 weeks. The patients’ demographic and oncological characteristics can be seen in **Tables 1**–**3**.

**Table 1:** Demographic and clinical characteristics of patients.

Variables	Category	N	%
Color/Breed	White	7	50
	Black	5	35,7
	Brown	2	14,3
Previous surgery	No	11	78,6
	Nodule excision	1	7,1
	Mastopexy	1	7,1
	Contralateral breast cancer	1	7,1
Systemic arterial hypertension	No	6	42,9
	Yes	8	57,1
Diabetes Mellitus	No	12	85,7
	Yes	2	14,3
Overweight/obesity	No	6	42,9
	Yes	8	57,1
Smoking	No	13	92,9
	Yes	1	7,1

**Table 2:** Tumor characteristics.

Variables	Category	N	%
Clinical Stage	IA	1	7,1
	IIA	4	28,6
	IIB	1	7,1
	IIIA	3	21,4
	IlIB	5	35,7
Skin involvement	No	9	64,3
	Yes	5	35,7
Localization	Upper outer quadrant	2	14,3
	Upper inner quadrant	7	50,1
	Union of upper quadrants	3	21,4
	Union of outer quadrants	1	7,1
	Union of inner quadrants	1	7,1
Tumor grade	I	1	7,1
	II	10	71,5
	III	3	21,4

**Table 3:** Adjuvant treatment and final results.

Variables	Category	N	%
Adjuvant Treatment			
Histology	Invasive breast carcinoma NST	12	85,7
	Invasive lobular carcinoma	1	7,1
	Invasive breast carcinoma NST + papillary	1	7,1
IHC	Luminal A	1	7,1
	Luminal B	8	57,2
	Triple negative	4	28,6
	Hybrid Luminal	1	7,1
Axillary surgery	Sentinel node biopsy	10	71,4
	Axillary dissection	4	28,6
Chemotherapy	No	1	7,1
	Neodiuvant	9	64,3
	Adjuvant	4	27,6
Endocrine Therapy	No	4	28,6
	Tamoxifen	3	21,4
	Aromatase inhibitor	7	50
Final Result			
Excelent/Good (Harvard Scale)	Early post-operative	12	85,7
	Late post-operative	11	78,6
Excelent/Good (BCCT.core)	Early post-operative	11	78,6
	Late post-operative	13	92,9

In our sample, no patient required ree-excision, there were no post-surgical complications and the hospitalization time of all patients was less than 24 hours.

Experts considered the breast symmetry results to be excellent/good for 85.7% of patients in the early post-operative period and 78.6% in the late post-operative period. Excellent/good ratings provided by the BCCT.core software amounted to 78.6% of cases in the early post-operative period and 92.9% in the late post-operative period.

The study found no statistically significant differences between the early and late post-operative results, whether using the Harvard scale or the BCCT.core software.

Out of all the ratings provided by the experts and the software in both periods, the rate poor was used only once, by an expert, for a case in the late pos-operative treatment.

According to the criteria of Landis and Koch, agreement between experts was rated as fair for the early post-operative period and moderate for the late post-operative period. Agreement between the Harvard scale and the BCCT.core software yielded identical agreement results.

Half of the patients rated their final symmetry as good; the other half rated it as excellent.

**Figure 3:**
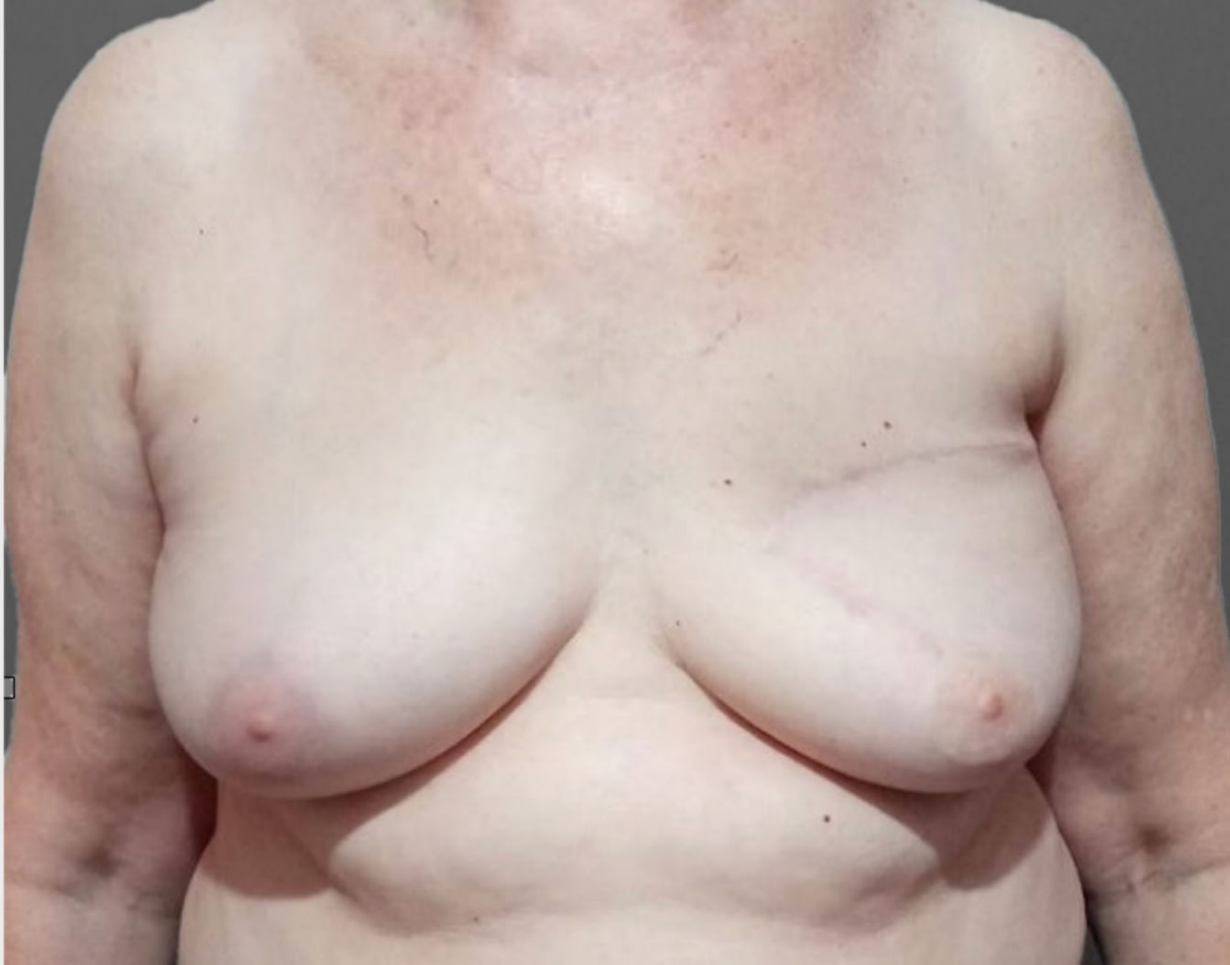
Late post-operative image of the same case.

**Figure 4:**
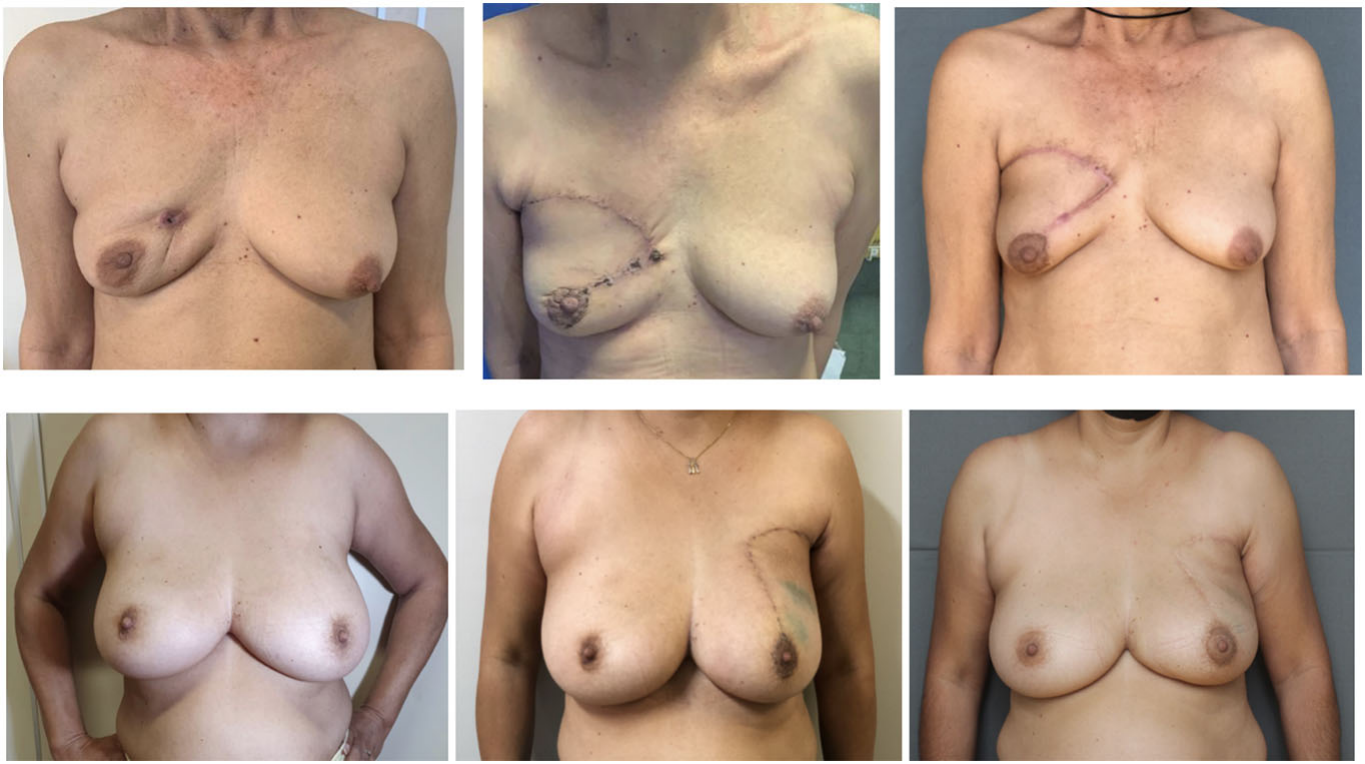
Preoperative, early post-operative and late post-operative images of typical cases treated using advancement-rotation flap technique.

**Figure 3** presents the late post-operative image of the case shown in **Figures 2**, **4** presents typical cases treated with the advance-rotation flap technique.

## Discussion

Oncoplastic surgery has been shown to be a safe and convenient option for patients requiring relatively large parenchyma or skin resections – or even for cases with challenging positions for resection closure (24, 25). The classical treatment for these patients would be a mastectomy or segmental resection, which would likely give rise to breast distortions and asymmetry. However, both options pose significant aesthetic consequences to patients.

For these patients, one option would be the oncoplastic surgical technique called mammoplasty with geometric compensation, which has been developed as an alternative to conventional conservative surgery, allowing for the resection of large tumors with skin resection in challenging locations such as the superior quadrants. This technique uses mammoplasty principles, therefore correcting breast ptosis and changing the shape and position of the nipple-areola complex. This would require contralateral surgery for symmetrization, which may be inconvenient in some situations (26, 27).

The proposed breast remodeling technique with dermoglandular advancement-rotation flap has made it possible to perform conservative surgery on relatively large tumors or even tumors requiring skin resection without the need for contralateral breast intervention. Unilateral surgery is faster and less morbid, an option that is especially beneficial for elderly women, patients with comorbidities, or even people who prefer to avoid bilateral surgery. None of the enrolled patients had any surgical complications.

In our sample, we observed a volumetric reduction of the operated breast in one pacient, associated with scar retraction after radiotherapy. In imaging tests performed, we observed areas of steatonecrosis that may explain what happened. Although this patient had undergone neoadjuvant systemic treatment, a 7 cm tumor was left for surgical approach. Due to the tumor size, a large dermoglandular advancement-rotation flap was necessary to correct the defect generated after removal of the tumor with free margins. Thus, this large area of ​​breast tissue that was detached from the pectoralis major muscle associated with the action of radiotherapy evolved with areas of steatonecrosis and fibrosis, generating an unfavorable final aesthetic result.

Our study did not assess scar patterns as a specific item, rather it was incorporated as a parameter for the breast symmetry assessment. Even though the dermoglandular advancement-rotation flap technique proposed in this study requires an extensive incision, its tension-free closure considerably decreases the probabilities of complications such as dehiscence and pathological scarring. The study also indicated that the scar issue had no negative impact on the final ratings provided either by the patients or the experts.

The advantage of this technique that we describe is that it allows a unilateral approach in those cases in which the the conventional breast-conserving surgery would not be suitable because of tumors being large, in unfavorable locations or involving or near skin. This technique allowed a quick surgery and with lower morbidity, what is specially important for patients with comorbidities, elderly or even those who do not want bilateral surgery. It also provides the possibility of carrying out conservative surgery in cases where a mastectomy would be performed, and allowed a satisfactory result, both from an oncological and aesthetic point of view.

In the evaluations carried out using the Harvard scale by 3 specialists, we found that 12 (85.7%) cases were categorized as Excellent/Good in the initial postoperative period and 11 (78.6%) of them remained with this evaluation in the late postoperative period. In 1 case there was a worsening of symmetry when comparing the two moments, a fact that we relate to the complications of radiotherapy. Two cases evaluated in the initial postoperative period as Fair/Poor remained so in the late postoperative period. Thus, the final surgical outcome was found to be Excellent/Good in 78.6% of the cases and Fair/Poor in 21.4%.

In the evaluation by the BCCT.core software, 11 (78.6%) cases were categorized as Excellent/Good in the initial postoperative period, 10 of these cases remained classified as such in the late postoperative period and there was a worsening of symmetry in 1 case, which was the same case mentioned above, in which the patient developed steatonecrosis. On the other hand, in the initial postoperative evaluation, 3 cases were categorized as Fair/Poor but all of them had an improvement in symmetry in the final postoperative evaluation, a fact that we can relate to the decrease in post-surgical edema and also due to the improvement in the quality of the photo taken correctly in a standardized fashion in the late postoperative period. The evaluation of the photo by the software can be hampered by poor positioning of the patient and poor image quality, impacting the result. Finally, evaluating the final surgical outcome using the BCCT.core software, we found 92.9% (13 cases) of Excellent/Good results and 7.1% (1 case) of Fair/Poor results.

The final outcome of the proposed technique amounted to an excellent/good rating of 78.6% according to the Harvard scale and 92.9% as per the BCCT.core software – figures that suggest that this technique can deliver satisfactory post-surgical symmetry results both according to subjective as well as objective criteria. These values are close to the ratings provided in the initial post-operative period, which demonstrates that satisfactory aesthetic results could already be seen in the initial post-operative period – an important aspect, especially at a time when patients are known to be emotionally vulnerable.

In our study, we identified that the percentage of cases evaluated as Excellent/Good by the Harvard scale applied by the specialists was lower than the percentage of cases that are in this same category by the evaluation of the BCCT.core software. One of the facts that could explain this difference would be the evaluation of the scar incorporated into the evaluation of symmetry which, when performed by specialists, has a more rigorous judgment than the software which, as we know through other studies, has a deficit in the evaluation of the scar.^29^

Patients’ self-assessments were also recorded and achieve excellent/good ratings in 100% of the cases. Some studies suggest that patients are likely to rate their own aesthetic results higher than the software or the expert panel (28). It is also known that patients’ self-assessments provide important information not only with respect to the aesthetic results of the breast, but also its functional aspects. Thus, more recent studies that included surgical outcome assessments have indicated that patients’ self-assessments should be carried out alongside expert panels and software assessments, since self-assessments reflect the patients’ psychological adaptation to both the aesthetic as well as functional aspects of the breast (28, 29).

The contrasting results arising from the different assessment methods makes a case for their complementarity and the importance of using and reporting distinct subjective and objective tools to assess the aesthetic outcomes of breast surgery (29).

## Conclusion

The dermoglandular advancement-rotation flap technique enables tumor resection with satisfactory margins and the correction of oncological defects in cases that are challenging due to location, size of the tumor or need for skin removal. Despite our small series, we found good results with oncological safety and it proved to be an effective technique to avoid mastectomy in selected cases.

Furthermore it provides good symmetry, as assessed both subjectively as well as objectively, and allows patients to undergo unilateral conservative surgery.

## Data availability statement

The original contributions presented in the study are included in the article/supplementary material. Further inquiries can be directed to the corresponding author.

## Ethics statement

Written informed consent was obtained from the individual(s) for the publication of any potentially identifiable images or data included in this article.

## Author contributions

MS contributed to the conception and design of the study, surgical procedures, use of software for symmetry assessment and statistical analysis, draft e wrote the manuscript. FB contributed to the conception and design of the study, surgical procedures, evaluations in postoperative appointments. JR contributed on surgical procedures, evaluations in postoperative appointments. TJ contributed on surgical procedures. VD contributed to the conception and design of the study, surgical procedures, evaluations in postoperative appointments. All authors contributed to the article and approved the submitted version.
